# Bio-Derived Fluorescent Carbon Dots: Synthesis, Properties and Applications

**DOI:** 10.3390/molecules27165329

**Published:** 2022-08-21

**Authors:** Manisha Kumari, Ganga Ram Chaudhary, Savita Chaudhary, Ahmad Umar, Sheikh Akbar, Sotirios Baskoutas

**Affiliations:** 1 Department of Chemistry and Centre of Advanced Studies in Chemistry, Panjab University, Chandigarh 160014, India; 2 Department of Chemistry, College of Science and Arts, and Promising Centre for Sensors and Electronic Devices (PCSED), Najran University, Najran 11001, Saudi Arabia; 3 Department of Materials Science and Engineering, The Ohio State University, Columbus, OH 43210, USA; 4 Department of Materials Science, University of Patras, 26504 Patras, Greece

**Keywords:** recycling, carbon dots, fluorescence, copper ion, sensing

## Abstract

The transformation of biowaste into products with added value offers a lucrative role in nation-building. The current work describes the synthesis of highly water-soluble, luminous carbon quantum dots (CQDs) in the size range of 5–10 nm from discarded rice straw. The small spherical CQDs that were formed had outstanding optical and luminescent qualities as well as good photostabilities. By performing quantitative multi-assay tests that included antioxidant activities, in vitro stability and colloidal assay investigations as a function of different CQD concentrations, the biocompatibility of CQDs was evaluated. To clearly visualize the type of surface defects and emissive states in produced CQDs, excitation-dependent fluorescence emission experiments have also been carried out. The “waste-to-wealth” strategy that has been devised is a successful step toward the quick and accurate detection of Cu^2+^ ion in aqueous conditions. The fluorescence-quenching behavior has specified the concentration dependency of the developed sensor in the range of 50 μM to 10 nM, with detection limit value of 0.31 nM. The main advantage of the current research is that it offers a more environmentally friendly, economically viable and scaled-up synthesis of toxicologically screened CQDs for the quick fluorescence detection of Cu^2+^ ions and opens up new possibilities in wastewater management.

## 1. Introduction

The presence of toxic contaminants, particularly heavy metal ions, has caused major health problems in humans, animals and other living things [1,2,3,4,5,6,7,8]. Out of a diverse range of existing metal ions, the toxic impact of copper (II) ions has adversely affected human health [2]. Copper ions (Cu^2+^) are the third substantial transition metal species found in the human body and play a significant role in various enzymes including tyrosinases, cytochrome c oxidase and many more [3,4]. Cu^2+^ ions also assist as a vital component for the functioning of different types of metalloenzymes present in living beings [5]. Cu^2+^ is one of the important dietary supplements required in the trace amount of 1.3 mg/day for the proper functioning of the human body. The extensive utilization of Cu^2+^ ions in the pharmaceutical, food supplements and agricultural sectors is the factor mainly responsible for its existence as a pollutant toxin in natural water resources [6,7]. As per the guidelines of the United States Environmental Protection Agency (USEPA), the permissible limit of Cu^2+^ in drinking water is around 1.3 ppm [8]. However, the excessive exposure of Cu^2+^ ions in the human body can lead to various health-related issues such as gastrointestinal disturbance, oxidative stress, Alzheimer’s disease, genetic disorders such as Wilson disease, Cancer, etc. [9,10]. The chronic impacts produced by the excessive accumulation of Cu^2+^ ions can even lead to kidney failure, liver damage and, in certain conditions, even death. Therefore, it becomes essential to devise advanced methodologies for the selective and sensitive detection of Cu^2+^ at very low concentrations for the betterment of human life and the surrounding environment [11].

In this scenario, a variety of commercially available analytical techniques such as atomic emission spectroscopy, mass spectroscopy, inductively coupled plasma-mass spectrometry (ICP-MS) and enzymes-based biosensors are employed for the detection of Cu^2+^ ions [12,13]. However, the high-cost instrumentation, multi-step sample preparation, tedious handling of instruments and improper real-time analysis have restricted the widespread usage of these techniques [14,15]. In addition to these commercial techniques, chemosensors-based methodologies have provided a better alternative for a simple, economical, fast and consistent way to detect the presence of Cu^2+^ ions in environmental samples. These chemosensors have shown notable changes in their optical or fluorescence signal after coordinating with specific metal ions. For instance, Gao et al. used the application of carbon dots derived from choline chloride-urea deep eutectic solvents and used it further for the detection of mercury ions, with a detection limit of 0.05 µm [16]. Jin et al. have also prepared two types of C-dots through solvothermal synthesis using sucrose or citric acid and tris(hydroxymethyl)aminomethane as precursor sources. The colorimetric method was developed for the detection of silver ions. The detection limit was evaluated to be 2.81 ppb [17]. The luminescence-based chemosensors have displayed q high selectivity and sensitivity towards these metal ions [18,19,20,21]. The inexpensive operational cost of these fluorescence-based sensors has further enhanced their application in metal ion sensing. Thus, the current work presents the economically viable and scaled-up approach for the detection of Cu^2+^ using bio-derived fluorescent CQDs from waste rice straw.

The scope of the existing waste-water management approach has been further expanded by the successful conversion of wasteful rice straw into value-added advanced C-dots using one-step thermal calcination at a low temperature. Around the world, the majority of agricultural byproducts are rice straws. About 234 million tons of rice straw waste are utilized each year in India alone for the production of fuel, bio-manure, animal fodder and bedding for livestock [22,23]. Small-scale farmers are now being forced to burn used rice straws to clear the fields due to the lack of accessible crop residue management for productive and sustainable agriculture [24]. By raising the risk of global warming and the excessive release of harmful gases into the atmosphere, burning has further harmed the ecosystem [25]. As a result, the current work, using leftover rice straw as a source of precursors for the synthesis of CQDs, has offered a better alternative to reusing leftover biomass, minimizing the production cost of CQDs and enhancing the sustainability of the environment through efficient crop residue management. The synthesis of bio-derived fluorescent CQDs has recently been the subject of several research studies [26,27]. Additionally, 5–24% lignin, 19–27% hemicellulose and 32–47% cellulose in rice straw have further reinforced its utilization as an effective precursor source for the synthesis of CQDs. The high specific surface area, outstanding optical and photoluminescence properties (with a high quantum yield of 66%) and superior biocompatibility has further enhanced its scope in metal ion-sensing applications [28]. In our view, the current work is among the primary testimonies that have been reported for the synthesis of CQDs from waste rice straw and which have employed them for metal ions sensing. The biocompatible aptitude of formed CQDs has further been evaluated by carrying out quantitative multi-assay studies including antioxidant activities, in vitro stability and colloidal assay studies as a function of different concentrations of CQDs. The current study has further enhanced the potential usage of formed CQDs in the medical field. The as-prepared CQDs have also exhibited a higher selective and sensitive aptitude towards Cu^2+^ ions in aqueous media. The importance of the developed system has further been augmented by carrying out the application of prepared CQDs for the testing of metal ion in real water samples. The current nanoprobe has provided several merits over those available in the literature. Therefore, the current work offers an innovative approach for recycling and reusing waste rice straw in CQDs and further using them for the identification of Cu^2+^ ions for wastewater treatment. 

## 2. Experimental Details 

### 2.1. Materials

The rice straw, as a precursor source for preparing CQDs, was collected from the village Rajpura, District Jind, Haryana, India and stored under dry conditions at room temperature. Hydrogen peroxide, sodium dihydrogen phosphate monohydrate and disodium hydrogen phosphate heptahydrate were acquired from Sigma Aldrich (>99 purity). The lyophilized form of Bovine Serum Albumin (BSA) and Human Serum Albumin (HSA) were purchased from HIMEDIA with 99% purity. Sodium sulphate, ammonium molybdate, iron chloride, potassium ferricyanide, histidine and zein protein of analytical grade with 99% purity were purchased from Sigma Aldrich. L-adrenaline was procured from HIMEDIA with 99% purity. NaOH pellets and concentrated HCl were obtained from Fischer Scientific with 99% purity. All of the respective biomolecules and metal ions used are: Ca^2+^, Hg^2+^, K^+^, Mg^2+^, Cu^2+^, Ni^2+^, Pb^2+^, Co^2+^, Zn^2+^, Fe^3+^, Al^3+^, glutathione, ascorbic acid, glutamic acid, citric acid, glycine and cysteine. They were purchased from Sigma Aldrich with 99% purity. All the used chemicals were of analytical grade and were utilized as such without any purification process. Deionized water was used for the preparation of solutions during the analysis. The various real water samples, i.e., tap water and rainwater, were obtained from Panjab University, Chandigarh, India, and the lake water was collected from Sukhna Lake, Chandigarh, India.

### 2.2. Bio-Derived Synthesis of Fluorescent C-Dots 

Biocompatible and highly photoluminescent CQDs were effectively fabricated via a one-step thermal calcination technique using non-food biomass, i.e., waste rice straw. At first, the rice straws were thoroughly rinsed with deionized water to eradicate dirt particles. The washed rice straws were then sun-dried before being chopped down and ground into pieces using a sanitized knife. The appropriately crushed rice straws were placed in a muffle furnace for 3 h of thermal calcination at a temperature of 320°C. The obtained brownish-black powder was then removed from the furnace and cooled down at room temperature. To generate fine particles, the prepared powder was thoroughly pulverized in an electric pestle mortar. Afterward, 2 g of the fine powder was dissolved in 200 mL of deionized water under constant stirring conditions for 24 h. The carbonized solution was then centrifuged for 10 min at 7000 rpm to remove the bigger particles. The resultant supernatant was filtered via a 0.22 m filter membrane to produce the pure form of CQDs.

### 2.3. Characterizations

An AICIL muffle furnace was used for the thermal calcination of the precursor source. The ultra-centrifugation was performed on the REMI-24 centrifuge. An IKA C-MAG HS7 magnetic stirrer was used for the stirring. The pH conditions of the samples were checked using a Mettler Toledo digital pH meter. A Panalytical X’Pert Pro X-ray diffractometer was used to investigate the structure and crystalline size of the synthesized CQDs. The structural morphology and size were further assessed using a High-Resolution Transmission Electron Microscopy (HRTEM) (Hitachi H7500, 100 kV) instrument. The optical properties were determined by using a Jasco V-750 UV-vis spectrophotometer in the wavelength range of 200–800 nm with a 1 cm quartz cell. The fluorescence emission profile was recorded in the Perkin Elmer LS 55 instrument in the working range of 200–800 nm. The quantum yield value of the CQDs was determined using the standard reference method by using a standard solution of quinine sulfate with a 54% quantum yield as a reference dye (λ = 310 nm, 0.1 M H_2_SO_4_ solution) [29]. The surface composition of the prepared CQDs has been analyzed using Fourier Transformed Infrared (FTIR) spectroscopy (Perkin Elmer RX1). The elemental composition and configuration of the synthesized C-dots have been determined using XPS (X-ray Photoelectron spectroscopy) (Physical Electronics, Model PHI 500 Versa Probe III). The purity and morphological variations were scrutinized utilizing Field Emission Scanning Electron Microscopy (FESEM, JEOL JSM 7500F). 

### 2.4. Toxicity Assessment of CQDs

#### 2.4.1. In Vitro Stability and Colloidal Assay of CQDs over Blood Components 

The in vitro assay has been chosen as an effective platform to recognize the stability of developed CQDs on the essential blood components in order to anticipate their future prospects in the medical field. Being one of the most vital components of living beings, different types of blood components, i.e., BSA, HSA, cysteine, histidine, L-adrenaline, zein and glutamic acid, have been chosen in this study to determine the effect of CQDs. For the experimentation, water was chosen as the standard reference solution. The respective solutions of 2% bovine serum albumin (BSA), 0.2 M cysteine and histidine were dissolved in 0.8% saline. Additionally, fresh phosphate buffer solutions were prepared in the pH range of 5.6, 6.2, 7.0 and 7.6 for diluting the samples. The corresponding effect of different concentrations of prepared CQDs (1000 ppm, 800 ppm and 500 ppm) was investigated to authenticate the biocompatibility of the prepared particles. Eventually, an equimolar mixture of prepared concentrations of CQDs and chosen blood components was allowed to incubate at 36°C for 48 h. Afterwards, the prepared samples were cooled down at room temperature, and their optical density values were determined by using a UV-vis spectrophotometer. Furthermore, the colloidal stability of the synthesized CQDs was investigated using human serum albumin (HSA), L-adrenaline, zein and glutamic acid. The respective samples were developed in phosphate buffer with a pH value of 7.4. Afterwards, different concentrations of prepared CQDs, i.e., 1000 ppm, 800 ppm and 500 ppm, were mixed in the equi-volume ratio (1:1) of HSA, L-adrenaline, zein and glutamic acid. The as-formed mixture was kept for incubation at 37 °C for 24 h [30,31] The supernatant of the respective solutions was taken out for the UV-vis analysis to observe the binding or colloidal permanency of the as-prepared CQDs with the chosen biological components. 

#### 2.4.2. Antioxidant Activities of CQDs

The antioxidant activities of CQDs have also been scrutinized to check for their reduction potential against free radicals and oxidation-inhibiting abilities. The respective study has provided direct confirmation of their biocompatible nature and has enhanced their potential in environmental remediation activities. In addition, this antioxidant assay has given direct evidence to ensure the free radical propagation abilities of CQDs in biological media [32]. Briefly, a total antioxidant capacity (TAC) reagent was formed by adding 4 mM ammonium molybdate and 28 mM sodium sulphate in 0.6 M H_2_SO_4_. Subsequently, different concentrations of CQDs (1000 ppm, 800 ppm and 500 ppm) were diluted with a methanol solution and then mixed with 3 mL of the TAC reagent. The resultant solution was set out for incubation at 95°C for 90 min. The standard blank solution was prepared by using deionized water. After cooling the samples for 15 min, the absorbance value of each sample was obtained at a wavelength of 680 nm in the UV-vis spectrophotometer. The ascorbic acid solution was set up as a positive control in the respective experiment.

The hydrogen peroxide scavenging ability of CQDs was also evaluated in this work. Briefly, 10 mM H_2_O_2_ solution was diluted in phosphate buffer saline (PBS) with a 7.4 pH value. Afterward, 1 mL of different concentrations of CQDs (1000 ppm, 800 ppm and 500 ppm) was added to 2 mL of H_2_O_2_ in PBS. The as-prepared solutions were allowed to incubate at 37°C for 10 min [33]. The reference solvent was measured against a blank (without hydrogen peroxide) solution. The percentage value of the hydrogen peroxide scavenging was calculated via the following equation:

(1) $$H_{2}O_{2}\;\mathrm{scavenging}(\%)=\left[\frac{A_{0}-A_{c}}{A_{0}}\right]\times 100$$

where A_0_ and A_c_ are the optical density values of the control and the samples, respectively. The inherent antioxidant potential of the prepared CQDs was also analyzed in terms of their electron acceptor and donor efficiency. For the analysis, three different concentrations of CQDs (1000 ppm, 800 ppm and 500 ppm) were mixed with 1 mL of deionized water. Afterward, the prepared solutions were diluted with 2.5 mL of 1% phosphate buffer (0.2 M, pH = 6.6) and 0.2 M potassium ferricyanide. The formed mixture was incubated at 50 °C for 30 min, and 2.5 mL of 10% trichloroacetic acid was added to the resultant mixture. The respective color changes in the incubated samples were observed. Afterwards, the respective solution was centrifuged at 2000 rpm for 5 min. The obtained supernatant was collected and diluted with 2.5 mL of deionized water and mixed with 0.1% of ferric chloride solution [34]. The absorbance value of the respective suspensions was measured at 700 nm for the analysis.

### 2.5. Sensing Aptitude of CQDs Using Fluorescence Studies

The sensing aptitude of the developed CQDs was tested against different types of metal ions and biomolecules such as Ca^2+^, Hg^2+^, K^+^, Mg^2+^, Cu^2+^, Ni^2+^, Pb^2+^, Co^2+^, Zn^2+^, glutathione, ascorbic acid, glutamic acid, citric acid, glycine and cysteine by using the standard fluorescence detection technique. For the experimentation, the equivalent volumes of metal ions and CQDs (1:1) were mixed together, and the fluorescence spectrum of an incubated equi-volume solution of each selected analyte molecule with CQDs in aqueous media was measured in the range of 200–800 nm. In addition, the detection sensitivity of the proposed sensor was evaluated by adding a fixed concentration of CQDs in the presence of different concentrations of metal ion. (Supplementary Materials)

### 2.6. Real Sample Analysis 

Four different types of environmental water sources comprising lake water, rainwater, tap water and deionized water were taken as real samples for the evaluation of the practical utility of the designed sensing system. For the respective study, the chosen samples were spiked with various concentrations of selective metal ions (i.e., Cu^2+^ = 5 μM, 15 μM, 25 μM, 35 μM and 45 μM) in the presence of CQDs. The respective emission spectra were recorded at the excitation wavelength of 310 nm. The recovery values were determined by the following equation:

(2) $$\mathrm{Recovery}(\%)=\left[\frac{C_{I}-C_{R}}{C_{x}}\right]$$

Here, C_I_ corresponds to the concentration of Cu^2+^ in real water samples after dilution with the standard solution of Cu^2+^, C_R_ refers to the concentration of Cu^2+^ in the targeted water sources before adding the external standard solution of Cu^2+^ and C_x_ is the amount of Cu^2+^ mixed in the water samples.

## 3. Results and Discussions 

### 3.1. Optical, Structural and Morphological Characteristics of CQDs 

**Figure 1:**
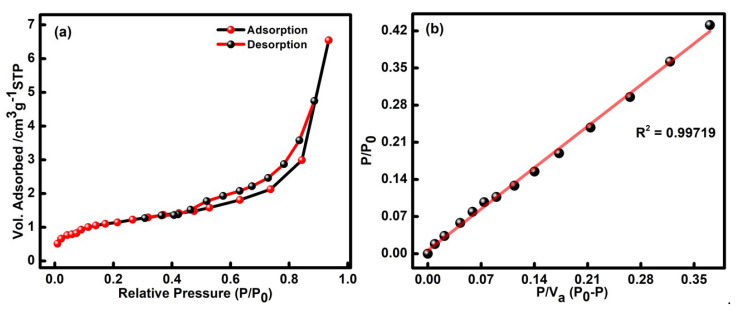
(**a**) Adsorption-desorption isotherm and (**b**) BET plot of developed CQDs.

The surface area of the CQDs is examined using N_2_ physio-sorption analysis in liquid nitrogen at 77 K, as presented in Figure 1a,b. The obtained adsorption-desorption isotherm of the formed CQDs is classified as a type IV isotherm with a hysteresis loop. For the examination, the saturated vapor pressure and adsorption temperature were set up at 100.95 kPa and 77 k. The surface area of the RS@CDs was determined to be 3.95 m^2^/g, as estimated in the BET plot (Figure 1b). Moreover, the respective linear fit curves displayed the mean pore diameter in the range of 10.23 nm. The pore size volume and micropore volume of the formed CQDs were found to be 0.01012 cm^3^/g and 0.9088 cm^3^/g, as estimated through the BET plot illustrated in Figure 1b. The respective outcomes have confirmed that the mesoporous nature of the developed particles can further be useful in detecting harmful pollutants to a large extent.

**Figure 2:**
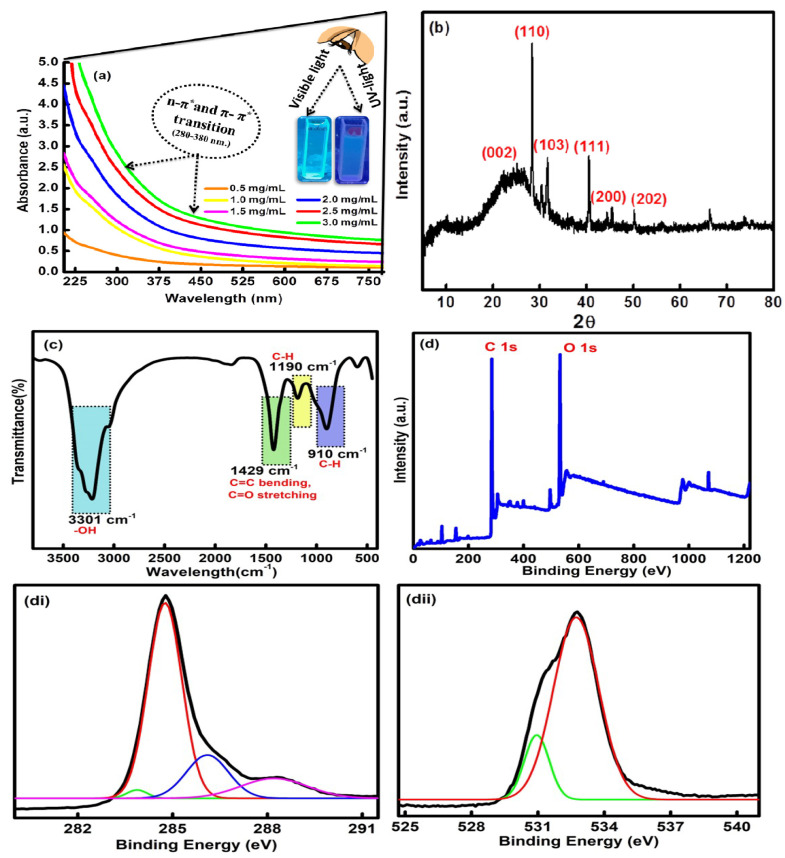
(**a**) Concentration-dependent UV-vis absorbance, (**b**) XRD, (**c**) FTIR and (**d**–**dii**) XPS survey scan of CQDs.

The UV-vis. spectral profile of the formed CQDs has displayed a characteristic absorption band between 300 and 400 nm (Figure 2a). The obtained absorption hump is related to the n-π* and π-π* transition of the C=O and C=C bond of the carbonyl and carboxyl groups on the exterior facade of the CQDs [35]. The synthesized CQDs have a reflected, milky, transparent color under the visible light, and an oceanic blue color was obtained under UV light (inset Figure 2a). The effect of different concentrations of CQDs, ranging from 1.0 mg/mL to 4.0 mg/mL, was also measured using UV-vis spectroscopic studies (Figure 2a). On interpreting the results, it was found that, with an increase in the concentration, three bands at wavelengths of 320 nm, 350 nm and 370 nm were observed, accompanied by a progressive increase in the absorbance of the samples. The results supported the idea that the absorbance of the sample was dependent on the concentration of the formed CQDs. The absorption band located around 320 nm is ascribed to the core state of the π-π* transition. The edge band of the n-π* transition was mainly seen at 350 nm in the prepared CQDs. However, the band at 370 nm mainly arises due to the existence of different types of functional groups such as carboxyl, carbonyl and hydroxyl bonded on the exterior edge of the CQDs [36]. The X-ray diffraction spectra of the fabricated CQDs have shown the existence of major peaks at 2θ = 23.5°, 27.29°, 29.8°, 40.1°, 45.2° and 49.9° corresponding to the (002), (110), (103), (111), (200) and (202) diffraction planes in the CQDs [37]. The presence of a broad peak at 23.5° indicates the carbonaceous core of the CQDs (Figure 2b). The outcomes have given direct evidence of the effective synthesis of CQDs from rice straw waste with a high purity and a good crystalline size ranging between 8 and 9 nm [JCPDS NO. 26-1076].

The surface composition of the CQDs has further been assessed by using FTIR analysis (Figure 2c). The spectra displayed three main sharp peaks at 3301 cm^−1^, 1429 cm^−1^ and 910 cm^−1^. However, two minor peaks were found at 1190 cm^−1^ and 610 cm^−1^. The high intense peak at 3301 cm^−1^ is mainly due to the presence of O-H stretching vibration from the hydroxyl groups over the exterior facade of the CQDs [38]. Further, the strong characterization peaks at 1429 cm^−1^ arose due to the presence of C=C bending and C=O stretching vibration in carbonyl and carboxyl functionalities on the surface of the CQDs. Another sharp peak at 910 cm^−1^ has been assigned to the C-H moieties in the CQDs [39]. The absorption peaks at 1190 cm^−1^ and 610 cm^−1^ appeared due to the C-H bending vibrations in the CQDs. The presence of hydroxyl, carbonyl and carboxyl functional groups on the outer surface of the CQDs has further enhanced their potential in sensing harmful toxic elements and has strengthened their aqueous solubility to a large extent [40].

The elemental configuration and content were further studied using XPS analysis. The high-resolution XPS survey has shown two typical peaks at 284.20 eV and 530.45 eV, which are further associated with the characteristic binding energy signals of C 1s and O 1s, respectively (Figure 2d). The core-level XPS resolution of C 1s indicates the existence of peaks at 284.84 eV, 285.50 eV, 286.54 eV and 287.12 eV, which were assigned to the C=C, C-C, C=O and C-O bonds (Figure 2di) [41]. The high-resolution XPS of O 1s was further deconvoluted into two peaks at 532.33 eV and 534.44 eV, which may be attributed to the C-O-C and C-OH functional groups in the as-synthesized CQDs (Figure 2dii) [42]. The results of the FTIR and XPS studies have further validated the existence of carbon and oxygen due to the presence of carboxyl, hydroxyl and carbonyl functional moieties over the exterior façade of the formed CQDs. 

**Figure 3:**
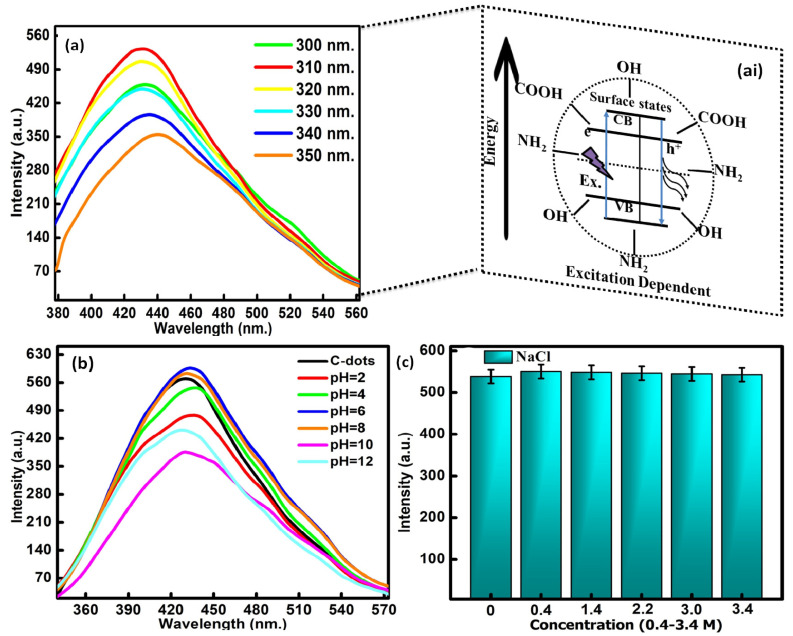
(**a**) Fluorescence emission spectra at different excitation wavelengths, (**ai**) mechanistic behavior of emission profile CQDs, (**b**) pH-dependent emission profile and (**c**) effect of ionic strength on the emission intensity of CQDs.

The excitation-dependent emission properties of CQDs were assessed to evaluate the stability of the formed particles (Figure 3a). The measurements were taken out as a function of the excitation wavelength in the range of 300 to 350 nm. The fluorescence spectrum has displayed the maximum emission intensity at 535 nm with the excitation wavelength of 310 nm. Therefore, all the fluorescence emission studies were carried out at a wavelength of 310 nm. These optoelectronic properties of the CQDs were associated with carbon core defects. The significant distribution of emissive trap sites in the CQDs is also responsible for the emission properties in the formed particles [43]. These surface defects have the ability to capture the epicenters of excitons due to the presence of oxygen and nitrogen-containing functional groups on the exterior facade of the CQDs. It will further enhance the surface-stated interrelated fluorescence in the developed CQDs. The quantum yield calculated using the reference standard method came out to be as high as 66% in reference to the quinine sulfate dye. The stability of the CQDs was further examined by varying the pH range from 2 to 4, 6, 8, 10 and 12 in the reaction media (Figure 3b). It was observed from the spectra that the emission intensity gradually increased in the pH range of 2 to 8, with the maximum emission at pH = 6. The results therefore supported the stability of the prepared particles in the slight alkaline medium. Upon enhancing the pH value of the reaction media above 8, a significant decrease in the peak position with the blue shift was observed in the fluorescence intensity (Figure 3c). This mainly arises due to the alterations in the fluorescence behavior of the acidic nature of the CQDs in the basic environment. The observed outcomes can be explained by the removal and addition of the proton part of the hydroxyl and carboxyl functionalities that exist on the exterior facade of the CQDs, which leads to the further modification of the electrostatic interacting behavior of the formed particles. Furthermore, the influence of ionic strength on the fluorescence potential of the CQDs has also been evaluated in the presence of different concentrations of NaCl solutions (0.4–3.4 M). The resultant graphs indicated that very high concentrations of NaCl produced a negligible effect on the fluorescence emission intensity of the formed particles (Figure 3c). The above outcomes have supported the stability of the formed particles in the presence of high-ionic-strength solutions. The outcomes have further supported the aptness of the formed particles in the field of toxin sensing because of their high emission and exceptional stability in different physiological and ionic strength media.

**Figure 4:**
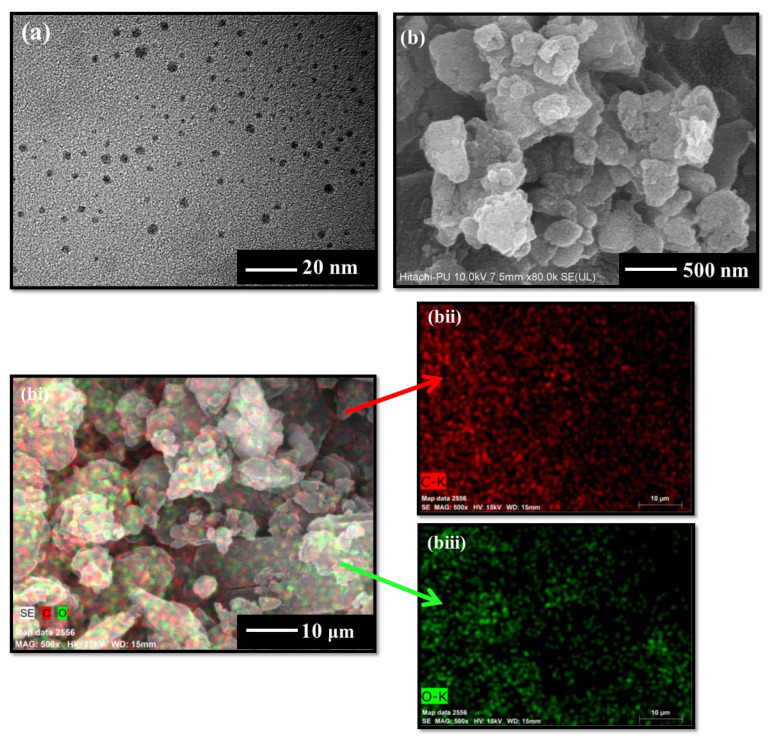
(**a**) HRTEM, (**b**) FESEM images and (**bi**–**biii**) elemental mapping of CQDs.

The morphological studies using HRTEM analysis have shown the existence of unevenly spherical and well-diffused particles with an average size range of 5–10 nm (Figure 4a). The FESEM images have further shown the existence of the circular lamellar porous nature of the CQDs (Figure 4b). However, the particles appeared to be in an agglomerated state in solid form, as shown in Figure 4b. The respective elemental analysis represents the main constituents of the formed particles containing the carbon (C) and oxygen (O) elements in the major atomic percentage, as illustrated in Figure 4bi-biii. These findings indicate that the developed CQDs have only specified elemental peaks, and the lack of any other peak indicates the high purity of the synthesized particles. 

### 3.2. In Vitro Stability and Colloidal Assay of CQDs over Blood Components 

**Figure 5:**
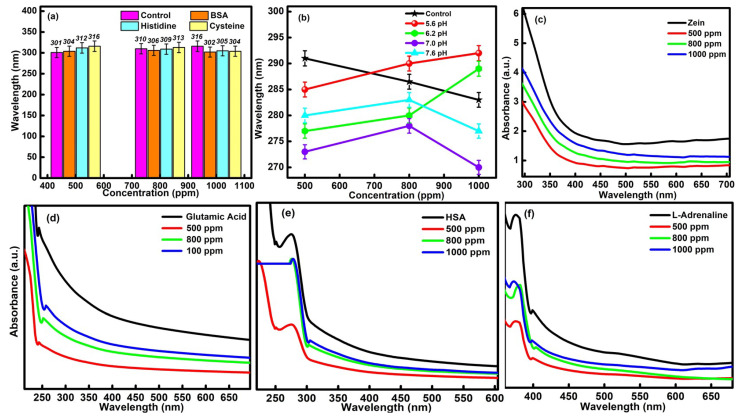
(**a**) In vitro stability activity in the presence of various essential blood elements (BSA, histidine and cysteine), (**b**) variations in the wavelength of C-dots in different pH buffer solutions (pH = 5.6, 6.2, 7.0 and 7.6) and the colloidal stability assay in the presence of (**c**) Zein, (**d**) Glutamic acid, (**e**) HSA and (**f**) L-adrenaline.

The prime requisite condition before executing the practical and in vivo application of formed CQDs is to evaluate their in vitro and colloidal stability with essential blood protein components. The respective study was performed by determining the absorption efficiency of the different types of blood components (BSA, HSA, cysteine, histidine, L-adrenaline, zein and glutamic acid) in the presence of different concentrations of CQDs (i.e., 1000 ppm, 800 ppm and 500 ppm). The corresponding effect of different pH conditions on the various biological components has also been tested for the developed CQDs. Upon interpreting the data, it was found that the high concentrations of CQDs have not produced any kind of visual or aggregation changes in the chosen blood components and supported the in vitro stability of particles in biological media. It was also observed that the wavelength of CQDs was only shifted in the permissible range of 14 nm in the presence of different blood components, which further confirmed the in vitro stability of CQDs (Figure 5a). The outcomes have further been compared with the buffer solutions of different pH values ranging from 5.6 to 7.6, (Figure 5b). The observed changes in the absorbance values were quite comparable with the earlier results. In addition, the slight hypochromic and bathochromic shift in the data is mainly attributed to the bio-compatible nature of the developed CQDs.

The colloidal stability of the CQDs was further assessed by carrying out the absorption studies in the presence of zein, glutamic acid, HSA and L-adrenaline (Figure 5c–f). The ascorbic acid has been used as a control during the measurements. The absorption peak at λ = 250 nm, in the case of glutamic acid, and the characteristic peak at λ = 310 nm, for HAS, have been checked in order to interpret the results. In the case of zein, the characteristic absorption peak was observed at λ = 350 nm. The UV-vis peak at λ =360 nm was checked in the presence of L-adrenaline. Upon increasing the concentration of CQDs from 500 ppm to 1000 ppm, a slight increment in the absorbance values was observed in the presence of all the chosen biological components. However, the highest absorbance value was discovered in the case of 1000 ppm of CQDs. This has been explained due to the maximum agglomeration of the particles of essential blood elements over the surface of CQDs. In addition, no negative effect of CQDs on the essential biological components has been observed, which further reflects the biocompatible nature of the prepared particles and made them useful in the biomedical field. Furthermore, the structure of synthesized particles does not show any kind of variations in the presence of the chosen blood components, which, in turn, supports their high in vitro stability and colloidal stability.

### 3.3. Antioxidant Activities of CQDs

#### 3.3.1. In Vitro Antioxidant Assay

**Figure 6:**
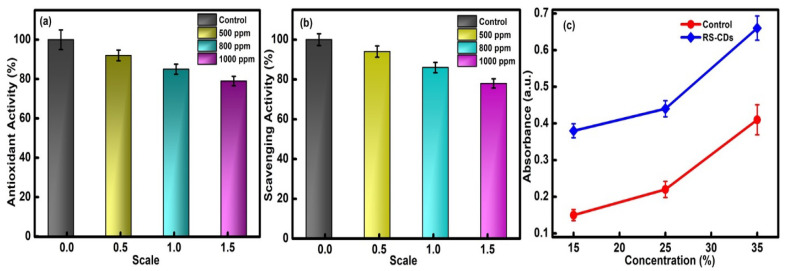
Effect of various concentrations of CQDs on (**a**) anti-oxidant activity, (**b**) hydrogen peroxide free radical scavenging assay and (**c**) total reduction capacity.

The antioxidant assay receives significant attention among researchers in the field of environmental and biological science. The basis of antioxidant activity deals with the reduced ability of free radical species in the presence of external moieties. The production of oxidative stress has further led to the generation of free radical species, which further inhibited the actions of DNA, proteins and lipids and is responsible for various types of disorders in living beings. However, the presence of antioxidant agents has produced a protective shield against the detrimental effect of free radical components in dose-dependent systems. Therefore, the efficiency of CQDs produced as an antioxidant agent has been tested as a function of concentrations ranging from 1000 to 500 ppm. The visual inspection of the solution in the presence of different concentrations of CQDs has shown the conversion of the transparent solution to a light greenish-blue color. This variation is mainly associated with the antioxidant nature of the formed particles. However, increasing the concentration of CQDs from 500 ppm to 1000 ppm has further reduced the antioxidant capacity of the chosen particles. However, the maximum antioxidant potential has been observed at 500 ppm of the CQDs (Figure 6a). This good performance is primarily explained by the presence of stable surface functional groups such as hydroxyl and carboxyl on the facade of the synthesized CQDs, which further helps to achieve the high anti-oxidant ability in formed particles.

#### 3.3.2. Hydrogen Peroxide Scavenging Ability of CQDs 

The free radical scavenging assay with hydrogen peroxide has also been checked to enhance the potential utilities of CQDs in the biomedical field. The absorbance values at λ = 290 nm were determined to apprehend the mechanistic behavior of the developed CQDs. Upon interpreting the results, a noticeable decrease in intensity was observed as a function of the concentration of RS@CDs ranging from 500 ppm to 1000 ppm in the presence of the H_2_O_2_ solution (Figure 6b). The results are comparable with the in vitro antioxidant assay. The slight decrease at high concentrations is mainly explained by the rupturing of peroxide bonding in the developed system. The existence of -OH groups over the surface of CQDs has the potential to inhibit the oxidative degradation of the prepared mixture solution. Therefore, the obtained results ensured the excellent scavenging activity of CQDs and enhanced their potential in biomedical applications. 

#### 3.3.3. Total Reduction Capacity of CQDs

The life cycle of different living beings is directly affected by the presence of free radical species. On account of this, it is essential to investigate the corresponding reduction efficiency of CQDs before giving any environmental exposure. During the experimentation, the obtained absorbance values were compared against the ascorbic acid solution chosen as the control standard. The optical density values at λ = 420 nm and 700 nm were checked as a function of different concentrations (1000 ppm, 800 ppm and 500 ppm) of CQDs. The linear increase in the absorbance value of the prepared mixture solutions was observed with an increase in the concentration of the CQDs, which supported the high reduction power of the formed particles (Figure 6c). The high reduction capability is due to the existence of hydroxyl groups over the external surface of the CQDs, which further enhanced the breaking of the weak peroxide linkage. The generation of the maximum reduction efficiency at a concentration of 500 ppm further confirmed the potency of the formed particles in the biological environment. 

### 3.4. Sensing Aptitude of CQDs 

**Figure 7:**
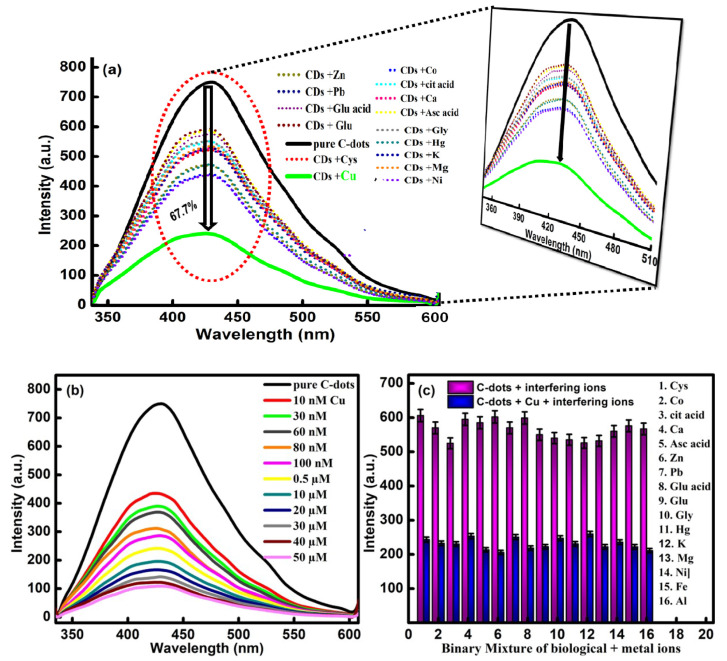
(**a**) Fluorescence emission spectra of CQDs in the presence of chosen metal ions and bio-molecules, (**b**) concentration variation of Cu^2+^ and (**c**) interference study of Cu^2+^ with several metal ions and biomolecules in the presence of CQDs.

The well-defined emission properties of CQDs were checked against various types of metal ions to enhance their scope in order to develop an easy-to-sense platform without any catalytic-mediated process. For the analysis, the changes in the fluorescence behavior of the developed CQDs have been compared in the presence of 15 different types of biomolecules and metal ions including Ca^2+^, Hg^2+^, K^+^, Mg^2+^, Cu^2+^, Ni^2+^, Pb^2+^, Co^2+^, Zn^2+^, glutathione, ascorbic acid, glutamic acid, citric acid, glycine and cysteine (Figure 7a). Upon comparing the data, it has been observed that the fluorescence intensity of the CQDs have generated small variations in the emission intensity. However, the presence of Cu^2+^ has produced maximum quenching in the emission intensity of CQDs. Therefore, the developed particles are found to be selective for Cu^2+^ ions. The quenching in the fluorescence emission intensity is explained by the presence of different types of functional groups over the surface of the CQDs, which further affected the optoelectronic properties via the non-radiative electron transfer mechanism. The turn-off fluorescence signal of the CQDs in the presence of Cu^2+^ ions is further explained by the splitting of the d-orbital of Cu^2+^ during the complex formation with the carboxyl and hydroxyl groups of the CQDs and Cu^2+^ [44]. As a result, electrons present in the excited state of the CQDs are transferred to the d-orbital of Cu^2+^. Additionally, the paramagnetic nature of the Cu^2+^ ion leads to the restraining of the transition of the electrons in the form of radiation emission, which further results in the fluorescence turn-off [45]. 

Furthermore, the sensitivity of the developed sensor has been probed by varying the concentration of Cu^2+^ (50 μm to 10 nm) via the titration-metric method (Figure 7b). The quenching efficiency of the CQDs has been enhanced significantly with the increase in the concentration of Cu^2+^ ions in the reaction media. The designed sensory probe has the potential to turn off the fluorescence intensity of the CQDs in the presence of Cu^2+^ ions, which further supports the high sensitivity and accuracy of the developed sensor.

To enhance the detection response of the CQDs, the selectivity response of the developed sensor has further been verified in the presence of different types of biomolecules and metal ions such as Ca^2+^, Hg^2+^, K^+^, Mg^2+^, Ni^2+^, Pb^2+^, Co^2+^, Zn^2+^, Fe^3+^, Al^3+^, glutathione, ascorbic acid, citric acid, glutamic acid, glycine and cysteine. The analysis has been performed by mixing the different metal ions with a 60 μM concentration of the Cu^2+^ ion. From the data, it has been observed that the fluorescence response of the sensor remains unaffected in the presence of different types of metal ions (Figure 7c). 

However, the presence of the Cu^2+^ ion has the potential to enhance the degree of quenching in the emissive response of the CQDs. The outcomes have suggested the high selectivity and sensitivity of the formed particles over any other metal ions and biomolecules. The complex formation between the carbonyl, carboxyl and hydroxyl groups of the CQDs with the Cu^2+^ led to the transference of electrons in the form of non-radiation recombination to achieve the selective quenching of Cu^2+^. The results gave direct evidence of the high selectivity and superior sensitivity of CQDs against Cu^2+^ ions. Furthermore, a better understanding of the quenching mechanism has been accessed by using the Stern–Volmer relationship [46].

F_0_/F = 1 + K_SV_[Q] (3)

**Figure 8:**
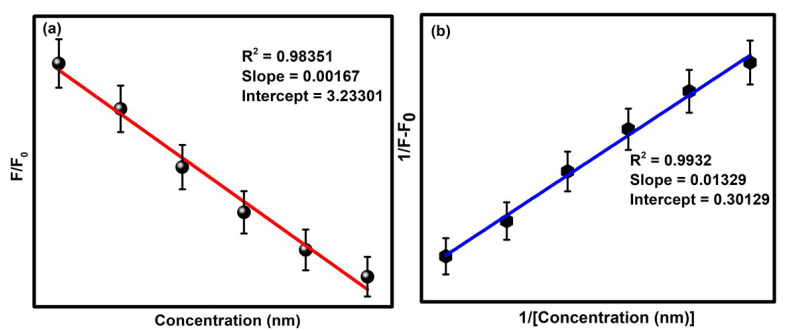
(**a**) Stern–Volmer plot and (**b**) B-H plot of CQDs in the presence of Cu^2+^ ions.

Here, F_0_ is the initial fluorescence intensity of the CQDs in the presence of the Cu^2+^ ion, whereas F represents the fluorescence intensity of the CQDs in the absence of the quencher Cu^2+^. Q refers to the concentration of Cu^2+^ in the solution, and K_SV_ is the Stern–Volmer constant. Significantly, the limit of detection was calculated by the standard deviation rule given below:

LOD = 3SD/P(4)

where SD indicates the standard deviation, and P is the slope of the Stern–Volmer plot (Figure 8a). Moreover, the binding efficiency of the developed sensory probe was estimated from the Benesi–Hildebrand plot (Figure 8b). The obtained values of the detection limit, binding constant, quantization constant and fluorescence quenching factor have been given in Table 1. The limit of detection of the sensor was calculated to be 0.31 nm.

### 3.5. Practical Application of CQDs for the Determination of Cu^2+^ in Real Water Samples

**Figure 9:**
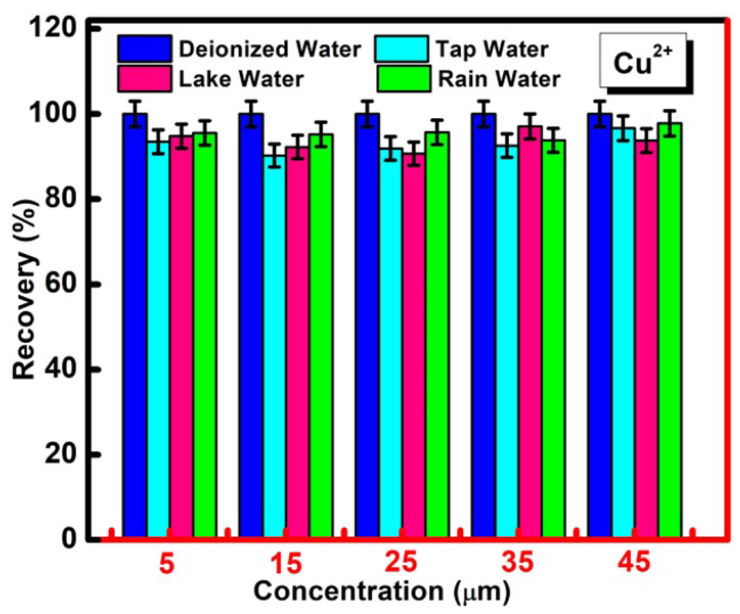
Percentage recovery of CQDs in the presence of different concentrations of Cu^2+^ in spiked samples of four different real water sources including tap water, rainwater, lake water and deionized water.

The practical utility of the developed nanoprobe was further accessed in real water samples taken from drinkable tap water, rainwater, lake water and deionized water. For the analysis, the synthesized CQDs were added to the different concentrations of Cu^2+^ (5 μm to 45 µm) in the chosen water samples, and their fluorescence intensity was measured via exciting the sample at a wavelength of 310 nm. The outcomes have confirmed the significant quenching in the presence of different concentrations of Cu^2+^ in each real water sample, which reflects the scope of application of the developed chemosensor for practical purposes. The recovery values were found to be in the range of 91–97% (Figure 9). The respective results have also displayed the feasibility of the synthesized CQDs for the practical detection of Cu^2+^ ions with a high sensitivity and specificity in different sources of water media. Moreover, the developed sensing strategy possessed a facile working principle in an economical manner. The upcycling of waste rice straw for the affordable synthesis of CQDs has enabled the green approach for the reproducible and precise determination of Cu^2+^ in real water sources.

## 4. Conclusions 

In conclusion, the present paper strategically used the waste-to-wealth approach for the successful transformation of waste rice straw into novel CQDs with superior optical and luminescence properties. The formed C-dots revealed outstanding photostability and amiability in different reaction environments. The different characterization methods via high-end sophisticated instruments have been performed for the physicochemical and structural authentication of formed CQDs. The formed CQDs have displayed a high aqueous solubility rate with a quantum yield value of 66%. The antioxidant assay has further been applied to check the biocompatibility of CQDs. Furthermore, the in vitro stability and colloidal stability have been determined in the presence of various essential biomolecules. Significantly, the fluorescence emission intensity of CQDs was around 67.7% quenched in the presence of Cu^2+^ ion. The current sensing was feasible in a one-step method in the absence of any surface modification. The low limit of detection (0.31 nm), effective selectivity and sensitivity, wide linear response (50 μm to 10 nm) and low-cost synthesis enabled CQDs as an advanced sensing tool for toxic Cu^2+^ ions. Moreover, CQDs were adequately used for their practical application in environmental water resources. This work not only enabled an opportunity for the fabrication of novel chemosensors via upcycling rice-straw waste but also presents an exceptional potential for the detection of Cu^2+^ ions.

## Figures and Tables

**Table 1:** The determined values of the detection limit, quantitation limit, binding constant, fluorescence recovery and quenching of synthesized C-dots.

S. No.	Parameter	CQDs
1.	Limit of detection (LOD)	0.31 nM
2.	Quantitation limit	2.22 nM
3.	Binding constant	0.42 nM
4.	Fluorescence recovery	0.75 nM
5.	Fluorescence-quenching factor	90–97%

## Data Availability

The data will made available on request.

## Supplementary Materials

The following supporting information can be downloaded at: https://www.mdpi.com/article/10.3390/molecules27165329/s1.
