# Supplementary material for: Bio-Derived Fluorescent Carbon Dots: Synthesis, Properties and Applications
Source: Molecules. 2022 Aug 21;27(16):5329. doi: 10.3390/molecules27165329 (PMC9416149; doi:10.3390/molecules27165329)
Supplement: Supplementary file 1 [file molecules-27-05329-s001.zip › molecules-1849152-supplementary.pdf]

Supplementary Information

Bio-derived Fluorescent Carbon Dots: Synthesis, Properties and Applications

Manisha Kumari [1], Ganga Ram Chaudhary [1], Savita Chaudhary [1,*], Ahmad Umar [2,3,*,†], Sheikh Akbar [3] and Sotirios Baskoutas [4]

1 Department of Chemistry and Centre of Advanced Studies in Chemistry, Panjab University, Chandigarh 160014, India
2 Department of Chemistry, College of Science and Arts, and Promising Centre for Sensors and Electronic Devices (PCSED), Najran University, Najran 11001, Kingdom of Saudi Arabia
3 Department of Materials Science and Engineering, The Ohio State University, Columbus, OH 43210, USA
4 Department of Materials Science, University of Patras, 26504 Patras, Greece

* Correspondence: chemsavita@gmail.com (S.C.); ahmadumar786@gmail.com (A.U.)

† Visiting Professor at Department of Materials Science and Engineering, The Ohio State University, Columbus, OH 43210, USA.

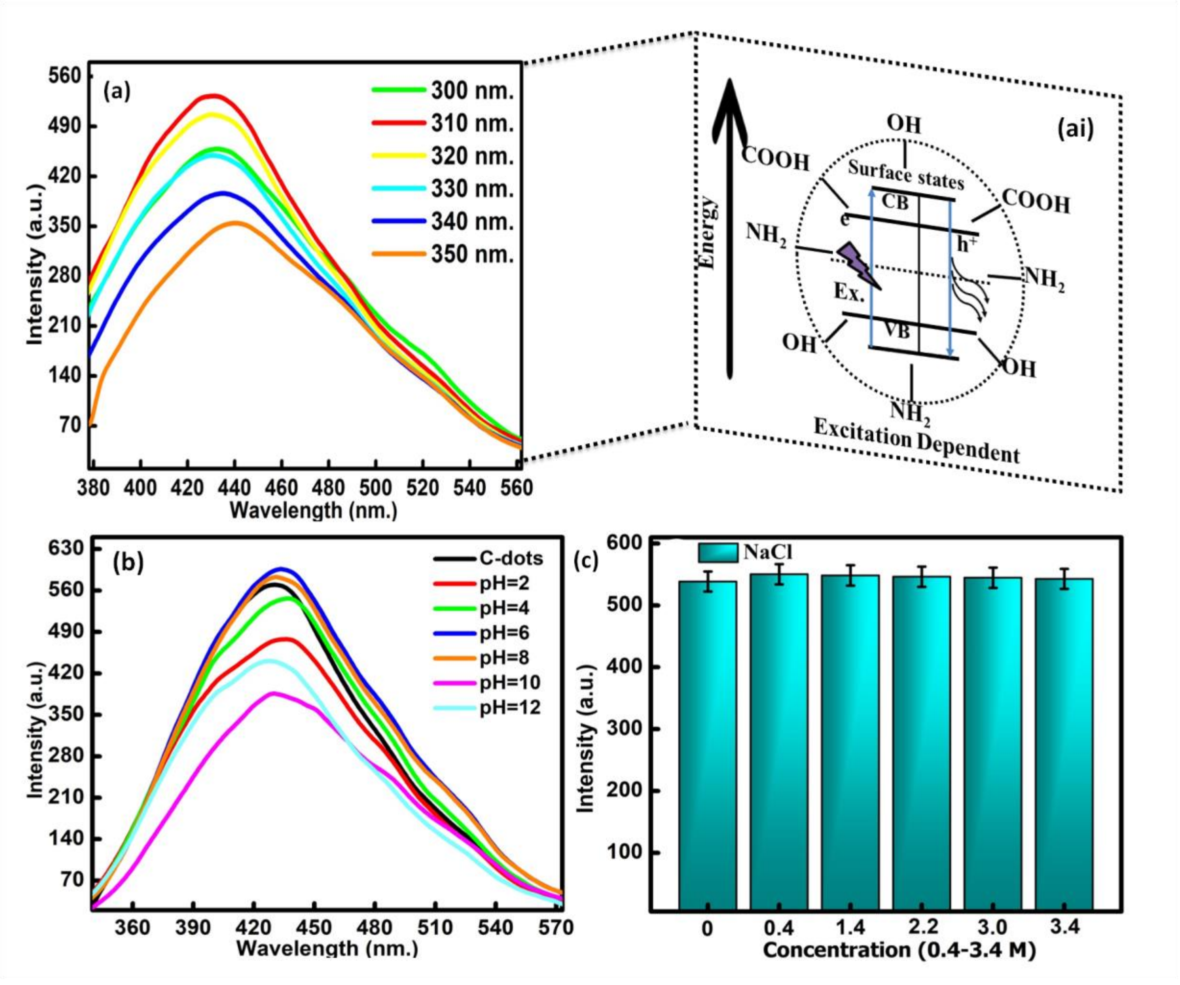

Figure. S1 (**a**) Fluorescence emission spectra at different excitation wavelengths, (**ai**) mechanistic behavior of emission profile CQDs, (**b**) pH-dependent emission profile and (d) effect of ionic strength on the emission intensity of RS@CQDs.
